# A Collection of Primary Tissue Cultures of Tumors from Vacuum Packed and Cooled Surgical Specimens: A Feasibility Study

**DOI:** 10.1371/journal.pone.0075193

**Published:** 2013-09-30

**Authors:** Laura Annaratone, Caterina Marchiò, Rosalia Russo, Luigi Ciardo, Sandra Milena Rondon-Lagos, Margherita Goia, Maria Stella Scalzo, Stefania Bolla, Isabella Castellano, Ludovica Verdun di Cantogno, Gianni Bussolati, Anna Sapino

**Affiliations:** 1 Department of Medical Sciences, University of Turin, Turin, Italy; 2 Department of Laboratory Medicine, Azienda Ospedaliera Città della Salute e della Scienza di Torino, Turin, Italy; 3 Institut Victor Babes, Bucharest, Romania; University Hospital of Modena and Reggio Emilia, Italy

## Abstract

Primary cultures represent an invaluable tool to set up functional experimental conditions; however, creation of tissue cultures from solid tumors is troublesome and often unproductive. Several features can affect the success rate of primary cultures, including technical issues from pre-analytical procedures employed in surgical theaters and pathology laboratories. We have recently introduced a new method of collection, transfer, and preservation of surgical specimens that requires immediate vacuum sealing of excised specimens at surgical theaters, followed by time-controlled transferring at 4°C to the pathology laboratory. Here we investigate the feasibility and performance of short-term primary cell cultures derived from vacuum packed and cooled (VPAC) preserved tissues. Tissue fragments were sampled from 52 surgical specimens of tumors larger than 2 cm for which surgical and VPAC times (the latter corresponding to cold ischemia time) were recorded. Cell viability was determined by trypan blue dye-exclusion assay and hematoxylin and eosin and immunohistochemical stainings were performed to appreciate morphological and immunophenotypical features of cultured cells. Cell viability showed a range of 84–100% in 44 out of 52 (85%) VPAC preserved tissues. Length of both surgical and VPAC times affected cell viability: the critical surgical time was set around 1 hour and 30 minutes, while cells preserved a good viability when kept for about 24 hours of vacuum at 4°C. Cells were maintained in culture for at least three passages. Immunocytochemistry confirmed the phenotype of distinct populations, that is, expression of cytokeratins in epithelioid cells and of vimentin in spindle cells. Our results suggest that VPAC preserved tissues may represent a reliable source for creation of primary cell cultures and that a careful monitoring of surgical and cold ischemia times fosters a good performance of primary tissue cultures.

## Introduction

Primary cultures represent an invaluable tool to set up functional experimental conditions that are instrumental to demonstrate biological mechanisms directly on human-derived tumor cells, however, creation of tissue cultures from solid tumors is troublesome and often unproductive particularly when dealing with primary cultures of carcinomas. Major biological issues are related to a relatively slow doubling time of epithelial cancer cells and to a rapid overgrowth with fibroblasts [1], depending also on the type of source lesions. In addition, technical aspects may affect the success rate of primary cultures in general. Such technical issues may stem from pre-analytical procedures routinely employed in surgical theaters and pathology laboratories. Indeed, two simple rules are mandatory for obtaining proper samples for cell cultures: (i) to acquire fresh specimens as soon as possible after completing the surgical procedure; (ii) to avoid both bacterial and fungal contamination of the specimens. The first rule matches with the need to reduce the ischemia process following the surgical procedure that stops with proper specimen fixation, since it allows activation of tissue enzymes, autolysis and degradation of proteins and nucleic acids [2], [3]. However, logistics management of specimens from the surgical theater to the pathology lab has to be considered with the priority that involves material handling, packaging and transportation. Depending on the hospital structure the transport of surgical specimens from the surgical theater to the pathology lab may prolong the ischemia time. In addition, transfer may be performed using the most variable types of boxes, transport media and at different temperatures and the interval between surgical intervention and sampling at the pathology laboratory is not monitored. A good model for preserving sterility and cell viability could be that proposed for preserving organ for transplantation, in which the first goal is reached by packing the organ in several layers of sterile containers and the second by cooling at 4°C the organ by surrounding it with an icy slush mixture [4]. In our University hospital we have recently introduced a new method of collection, transfer and preservation of surgical specimens [5], [6], [7], [8]. This method requires immediate vacuum sealing of excised specimens at surgical theaters, followed by time-controlled transferring at 4°C to the pathology laboratory [6], [7]. Such a procedure allows for having fresh (i.e. not fixed) tissues and it has been demonstrated not to affect morphology and to best preserve nucleic acids (DNA and RNA) and proteins [6], [7], [9].

In this study we tested the feasibility of setting up primary cell cultures derived from vacuum packed and cooled (VPAC) preserved tissues and to evaluate their performance and success rates.

## Materials and Methods

### Reagents

Tissue samples for cell cultures were collected in RPMI 1640 serum free medium, supplemented with 1% penicillin-streptomycin-fungizone.

The basal media used for cell culture was a mixture of DMEM (Dulbecco’s Modified Eagle Medium) and F12 in 1∶1 proportion. For preparation of complete media, 10% fetal bovine serum (FBS), 1% L-glutamine and 1% penicillin-streptomycin-fungizone were added to the basal media mixture. The complete media was supplemented with 10 ng/mL human epidermal growth factor (EGF), 5 µg/mL insulin and 400 ng/mL hydrocortisone. For breast cancer samples, the complete media contained also 5 ng/mL 17-beta-estradiol and 500 ng/mL progesterone.

For enzymatic cell dissociation Collagenase Type IV was used while trypsin-EDTA was used for passaging. All reagents were from Sigma-Aldrich, St Louis, MO, USA.

All materials used in this experiment were sterile to prevent contamination.

### Vacuum Sample Collection

The study was approved by the ethic institutional review board for “Biobanking and use of human tissue for experimental studies” of the Pathology Services of the Azienda Ospedaliera Città della Salute e della Scienza di Torino. Written informed consent was obtained from all patients for their tissue to be used in research. Following excision in the surgical theater, surgical specimens were immediately placed into beta-ray sterilized plastic bags and vacuum sealed using the TissueSAFE machine (Mod. VAC 10, by Milestone, Bergamo, Italy; see www.milestonemedsrl.com), according to a procedure originally reported by our group [7] and currently implemented as a standard technique in our hospital [5], [8]. The VPAC specimens were kept and transferred to the pathology lab at 4°C in a chilled plastic box. Once in the lab, tissues were kept in a refrigerator at 4°C until processing. The surgical and VPAC times of the sample and the histotype, the percentage of stroma and epithelial cells of sample received from surgical theaters were recorded in a dedicated database.

As a separate analysis, two large specimens of reduction mammoplasty were divided in three parts (each of approximately 10×5×5 cm) in order to evaluate the decrease of temperature by using a digital thermometer with stainless steel sensor probe at different conditions of storage (i.e. vacuum sealed at room temperature, vacuum sealed at 4°C, not vacuum sealed at 4°C).

### Cell Culture Procedure

The study was conducted on surgical specimens of tumors larger than 2 cm, leading to a cohort of 52 surgical samples, including 13 colorectal carcinomas, 6 lung carcinomas, 27 breast carcinomas, 2 adrenocortical adenomas, 3 gastric carcinomas and 1 thyroid carcinoma (see Table 1 for details). Samples for cell cultures were collected from “left over tissues” (i.e. tissue residuals not used for diagnostic and therapeutic purposes) by using sterile scalpels. Samples of 1×1 cm around 0.5 cm in thickness were collected in sterile tubes containing 10 mL of RPMI serum free medium, supplemented with 1% penicillin-streptomycin-fungizone. Tissue samples were washed 3 times in 20 mL of the same medium, then finely minced by surgical blades into approximately 1×1 mm fragments and divided in two aliquots. One aliquot was processed for cryopreservation, while the other tissue fragments were incubated at 37°C with collagenase type IV (1 mg/mL; 1∶1 RPMI, final volume 10 mL), for 3–5 hours until complete disaggregation of fragments was obtained. Digested samples were shaken vigorously by hand to disaggregate possible residual large clumps. Collagenase activity was blocked by addition of 10 mL of RPMI with 10% FBS. After centrifugation at 800 rcf for 6 minutes, the cell pellets were re-suspended in complete culture medium. The final cell suspension was seeded in Petri dishes as passage 0 and kept in a humidified incubator with 5% CO_2_ at 37°C. Culture medium was changed first at the time of cell attachment and, subsequently, three times a week. Cell growth was monitored daily in flasks with an EVOS inverted microscope (Advanced Microscopy Group, Bothell, WA, USA) during the first week. The morphology of the cell population (spindle or mixed spindle-epithelioid) was recorded.

**Table 1:** Details of the 52 source lesions for cell cultures included in the study, correspondent surgical, and VPAC times of the specimens and percentage of viable cells in the primary cultures.

				Tumor histology			Cell culture	
#	Organ	Surgical Time [hours (h), minutes (′)]	VPAC Time [hours (h), minutes (′)]	Histological Type	% Stroma	% Tumor Cells	% CellViability	Cell Population
**1**	Colon	1h45′	3h45′	ADC	30	70	**0.0%**	/
**2**	Stomach	2h15′	4h10′	ADC	20	80	**0.0%**	/
**3**	Lung	1h20′	4h15′	ADC	40	60	**95.3%**	Spindle
**4**	Lung	1h20′	4h30′	ADC	40	60	**93,9%**	Mixed
**5**	Stomach	1h30′	4h45′	ADC	35	65	**97,0%**	Spindle
**6**	Colon	1h10′	5h00′	ADC	15	85	**100,0%**	Mixed
**7**	Colon	0h40′	5h25′	ADC	5	95	**96,8%**	Mixed
**8**	Colon	1h15′	5h25′	ADC	30	70	**92,2%**	Mixed
**9**	Adrenal	1h20′	5h25′	ADENOMA	10	90	**86,1%**	Spindle
**10**	Breast	1h35′	20h40′	ILC	40	60	**0,0%**	/
**11**	Lung	2h15′	21h20′	SFT	8	92	**96,4%**	Mixed
**12**	Breast	0h45′	21h40′	IC-NST	25	75	**93,6%**	Mixed
**13**	Breast	1h40′	21h43′	IC-NST	10	90	**94,8%**	Mixed
**14**	Breast	0h20′	22h20′	ILC	8	92	**98,8%**	Mixed
**15**	Breast	0h55′	22h20′	IC-NST	15	85	**95,0%**	Mixed
**16**	Colon	1h10′	22h30′	ADC	30	70	**92,2%**	Spindle
**17**	Lung	0h10′	22h35′	ADC	2	98	**97,7%**	Mixed
**18**	Lung	1h20′	22h35′	SC	50	50	**96,5%**	Mixed
**19**	Colon	1h20′	22h45′	ADC	40	60	**93,5%**	Spindle
**20**	Breast	1h10′	23h10′	IC-NST	20	80	**96,9%**	Mixed
**21**	Breast	0h11′	23h17′	IC-NST	50	50	**96,6%**	Mixed
**22**	Breast	1h50′	23h20′	IC-NST	60	40	**96,3%**	Mixed
**23**	Breast	0h25′	23h25′	ILC	50	50	**96,1%**	Mixed
**24**	Breast	1h10′	23h46′	IC-NST	40	60	**98,5%**	Mixed
**25**	Breast	0h05′	23h50′	IC-NST	30	70	**95,9%**	Mixed
**26**	Colon	0h45′	23h50′	ADC	15	85	**86,6%**	Mixed
**27**	Breast	1h10′	23h50′	MC	10	90	**94,0%**	Mixed
**28**	Breast	1h20′	24h00′	IC-NST	55	45	**84,0%**	Spindle
**29**	Breast	0h50′	24h10′	IC-NST	30	70	**97,9%**	Mixed
**30**	Breast	0h25′	24h16′	IC-NST	10	90	**99,3%**	Mixed
**31**	Breast	0h15′	24h30′	IC-NST	20	80	**99,2%**	Mixed
**32**	Breast	1h10′	24h35′	IC-NST	65	35	**0,0%**	/
**33**	Breast	0h40′	25h00′	PDC	30	70	**96,5%**	Mixed
**34**	Lung	1h40′	25h00′	ADC	40	60	**97,3%**	Spindle
**35**	Breast	0h50′	25h05′	ILC	40	60	**96,7%**	Mixed
**36**	Breast	0h35′	25h20′	IC-NST	30	70	**96,0%**	Mixed
**37**	Colon	1h45′	26h10′	ADC	40	60	**98,6%**	Spindle
**38**	Thyroid	2h20′	26h40′	HCC	20	80	**96,3%**	Mixed
**39**	Colon	1h45′	27h40′	ADC	25	75	**90,7%**	Spindle
**40**	Breast	0h27′	28h20′	ILC	30	70	**0,0%**	/
**41**	Colon	0h50′	29h00′	ADC	20	80	**86,5%**	Mixed
**42**	Colon	1h25′	39h20′	ADC	20	80	**0,0%**	/
**43**	Breast	0h50′	42h15′	IC-NST	40	60	**89,0%**	Mixed
**44**	Breast	0h20′	43h05′	ILC+IPC	25	75	**97,3%**	Mixed
**45**	Breast	1h00′	45h05′	IC-NST	80	20	**98,9%**	Spindle
**46**	Breast	3h00′	45h50′	IC-NST	40	60	**98,7%**	Mixed
**47**	Breast	0h40′	46h47′	IC-NST	20	80	**96,3%**	Mixed
**48**	Colon	1h10′	70h15′	ADC	30	70	**97,5%**	Spindle
**49**	Breast	2h25′	72h25′	ILC	20	80	**95,5%**	Mixed
**50**	Colon	1h45′	73h45′	ADC	25	75	**96,7%**	Spindle
**51**	Adrenal	3h45′	74h05′	PHEO	5	95	**0,0%**	/
**52**	Stomach	1h30′	75h15′	ADC	52	48	**0,0%**	/

Legend: ADC: adenocarcinoma; HCC: Hurtle cell carcinoma; IC-NST: invasive carcinoma of no special type; ILC: infiltrating lobular carcinoma; IPC: intracystic papillary carcinoma; MC: medullary carcinoma; PDC: poorly differentiated carcinoma; PHEO: pheochromocytoma; SFT: solitary fibrous tumor; SC: squamous carcinoma.

Whenever the cell pellet was adequate, cells were seeded in another Petri dish containing sterilized coverslips (22×22 mm) to carry out Hematoxylin and Eosin (H&E) staining and immunocytochemical (ICC) reactions. Each time cells grown at confluence were split was considered a new passage.

### Viability Assay and Cryopreservation of Cells

Viability of cells was determined by trypan blue dye (0.4% in PBS)-exclusion assay. Aliquots of cell suspension were incubated with trypan blue solution (1∶1) for 5 min. Finally cells were transferred to the Burker chamber and counted by light microscope. Dead cells were defined as those stained with the dye. The percentage of living cells was calculated by the relationship between the number of viable cells and the total number of cells counted. Aliquots of cells were cryopreserved into sterile cryo-tubes in 1.5 mL freezing medium (FBS containing 10% DMSO). Tubes were kept at −80°C (for a maximum of 6 weeks) or, following overnight cooling, transferred into liquid nitrogen (for longer storage periods).

### H&E Staining and ICC Reactions

To evaluate the proportion of stroma and tumor cells in the sample collected for primary cultures we examined the H&E histological slide obtained from sampling of a parallel tumor area. To evaluate morphology of cultures, cells were grown on coverslips (sealed on a standard slide) and washed in phosphate-buffered saline (PBS 1X) for 5 minutes, fixed in 4% neutral-buffered formalin for 10 minutes and dehydrated through a series of alcohols up to absolute alcohol and stained with H&E staining.

If the culture showed a mixed population we tried to characterize the cells using specific organ related markers. ICC was performed using an automated slide processing platform (Ventana BenchMark XT AutoStainer, Ventana Medical Systems, Tucson, AZ, USA). The following primary antibodies were used: mouse anti-pancytokeratin (clone AE1-AE3-PCK26, Ventana; pre-diluted, antigen retrieval: Protease 1 (Ventana) for 4 minutes), anti-vimentin (clone R9, Dako, Glostrup, Denmark; dilution 1∶50, antigen retrieval: pre-diluted pretreatment solution Cell Conditioning 1 (CC1, Ventana) for 20 minutes), anticytokeratin-19 (clone NCL-CK19, Leica Novocastra; dilution 1∶50, antigen retrieval: CC1 36 for minutes), anti-cytokeratin-14 (clone NCL-LL002, Leica Novocastra; dilution 1∶100, antigen retrieval: CC1 for 36 minutes), anti-cytokeratin-7 (clone OVTL, Dako, Glostrup, Denmark; dilution 1∶100, antigen retrieval: CC1 for 36 minutes) and anti-TTF1 (clone 8G7G3/1, Roche Diagnostics; pre-diluted, antigen retrieval: CC1 for 36 minutes).

### Statistical Analysis

Results were analyzed by contingency tables using the Fisher’s exact test. Paired Student’s T-test was used for continuous variables. A p value <0.05 was considered statistically significant.

## Results

### Effect of Vacuum and Cooling on Specimen Temperature

**Figure 1:**
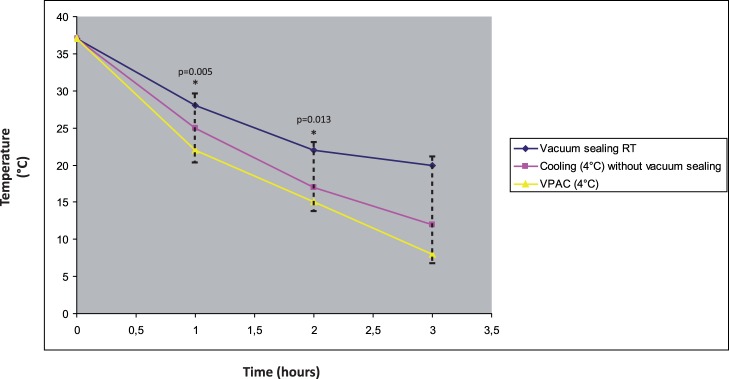
Effect of vacuum and cooling on specimen temperature. Results of temperature monitoring over time of a specimen of reduction mammoplasty. The specimen was subdivided in three parts that were stored under three distinct conditions, i.e. vacuum sealed at room temperature (RT), vacuum sealed at 4°C, cooled at 4°C without vacuum sealing. VPAC produced a more rapid decrease of temperature than non-VPAC procedures, while vacuum sealing at room temperature did not significantly affect temperature decrease. The * indicates statistically significant differences (p = 0.005 at 1 hour, p = 0.013 at 2 hours).

VPAC produced a more rapid decrease of temperature as compared to non-VPAC procedures, while vacuum sealing at room temperature did not significantly affect temperature decrease (Figure 1). Statistically significant differences between groups were observed at 1 hour and 2 hours (p = 0.005 and p = 0.013, respectively). The results were consistent for the whole set of samples obtained from two different reduction mammoplasty specimens.

### Cell Viability and Influence of Surgical and VPAC Time

**Figure 2:**
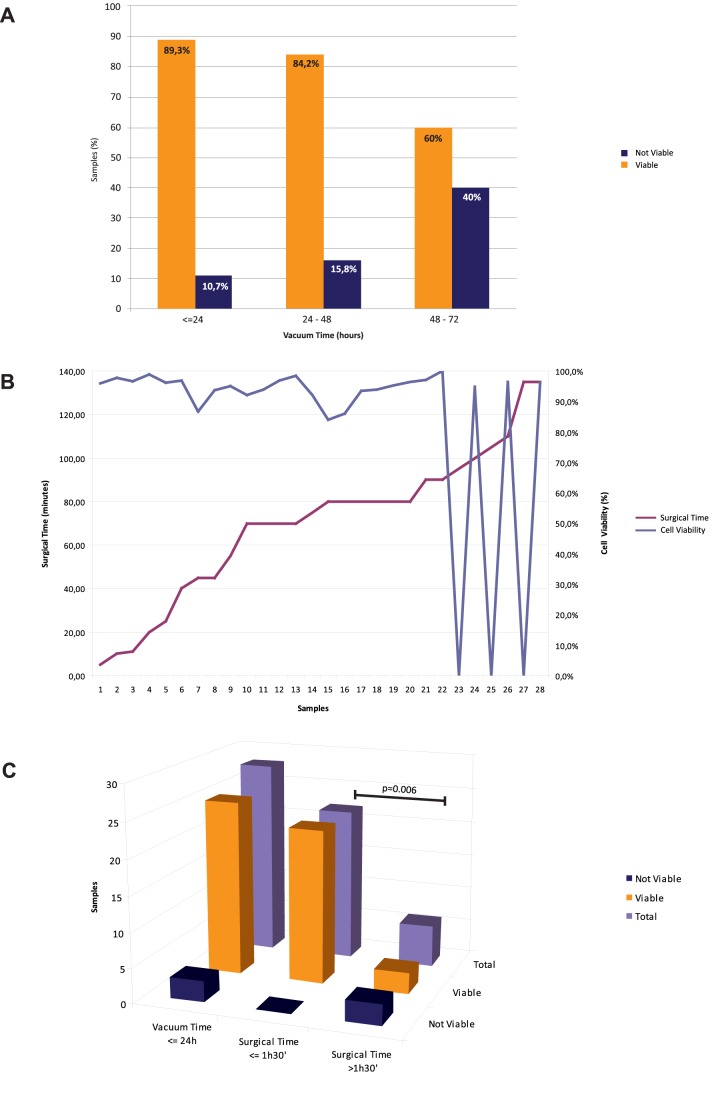
Reciprocal relationship between surgical and VPAC times with cell viability. A: cell viability was 89.3% in specimens with VPAC ≤24 h, 84.2% in specimens with 24 h<VPAC≤48 h and 60% in specimens with VPAC >48 h. B: The critical surgical time was set around 1 hour and 30 minutes, as shown by the drop of cell viability line as compared to the surgical time. This analysis was performed in the subset of specimens with VPAC time ≤24 h (in order not to introduce a bias due to long cold ischemia time). C: Assessment of cell viability in specimens with VPAC time ≤24 h and comparison with surgical time. The histogram shows the number of viable and not viable cell cultures obtained from samples with a VPAC time ≤24 h: total number of specimens with VPAC time ≤24 h on the left, specimens with VPAC time ≤24 h and surgical time ≤1 hour and 30 minutes in the middle, specimens with VPAC time ≤24 h and surgical time >1 hour and 30 minutes on the right. The difference in cell viability between the two groups (surgical time <1 hour and 30 minutes and surgical time >1 hour and 30 minutes) was statistically significant (p = 0.006, Fisher’s exact test).

Table 1 lists the details of 52 collected cases (site of lesion origin, histotype and percentage of stromal and tumor components) with correspondent surgical time (i.e. the time between the beginning of surgery - incision of the skin - and the surgical removal of tissues) and VPAC time (i.e. time between surgical removal of tissues and their fixation, i.e. “cold ischemia time”). Both surgical and VPAC times were highly variable (Table 1). For 28 cases the VPAC time was 0< hours ≤24, for 19 cases it was 24< hours ≤48, for 2 cases it was 48< hours <72, for 3 cases it was >72 hours. Establishment of short-term primary cultures was successfully achieved in 85% of processed samples (44 out of 52), regardless of the specimen size and origin and cellularity of source lesions. Only one case of gastric cancer developed bacterial infection after one day of culture (Table 1, case #2). Cell viability ranged from 84 to 100% (mean: 95.2%) in the 44 obtained primary cultures. Length of both surgical and VPAC times affected cell viability. Cells preserved a good viability when kept for about 24–48 hours of VPAC at 4°C (Figure 2A), while the critical surgical time was set around 1 hour and 30 minutes (Figure 2B). More specifically, the cut-off time of 1 hour and 30 minutes for surgical time was data-driven as follows: first, we focused on 27 samples with VPAC time ≤24 h, in order not to introduce a bias due to a long cold ischemia time. Within such a subgroup we found that with the increased surgical times cell viability was maintained (≥84%) up to the cut-off time of 1 hour and 30 minutes (Figure 2B). Beyond that period, cell viability dropped to 0% in 3/6 cases, two of which had VPAC time shorter than 5 hours (Table 1 case #1 and #2). The difference in cell viability between the two groups (surgical time <1 hour and 30 minutes and surgical time >1 hour and 30 minutes) was statistically significant (p = 0.006, Fisher’s exact test) (Figure 2B).

Then, we analyzed the influence of VPAC time on cell viability in more in details. Cell growth was not feasible in 10.7% of specimens with VPAC time ≤24 hours, in 15.8% of tissues with 24< hours ≤48 VPAC time and in 40% of samples with >48 hours VPAC time (no statistically significant differences between the groups) (Figure 2A). However, the examination of the corresponding histological samples showed preservation of morphology regardless of surgical and VPAC times.

### Characterization of Cell Primary Cultures

**Figure 3:**
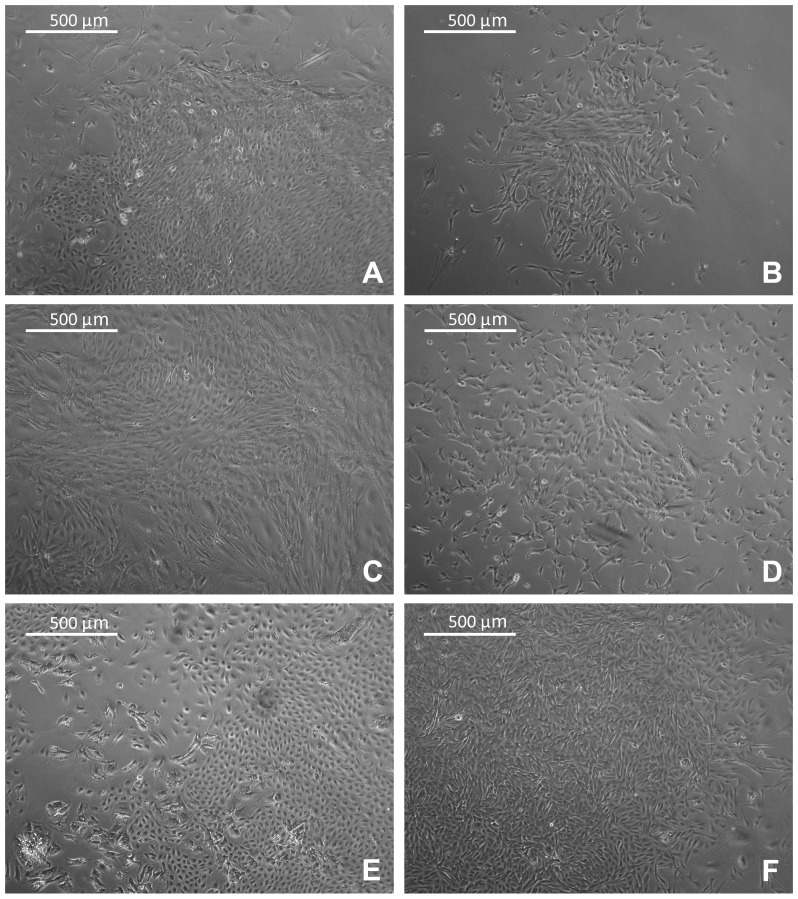
Primary cell cultures in flasks. EVOS inverted microscope images (4× magnification) of primary cell cultures of tumors from different organs growing adhering to the flasks. A: breast carcinoma; B: colorectal carcinoma; C: lung adenocarcinoma; D: gastric carcinoma; E: pheochromocytoma; F: Hurtle cell carcinoma of the thyroid.

**Figure 4:**
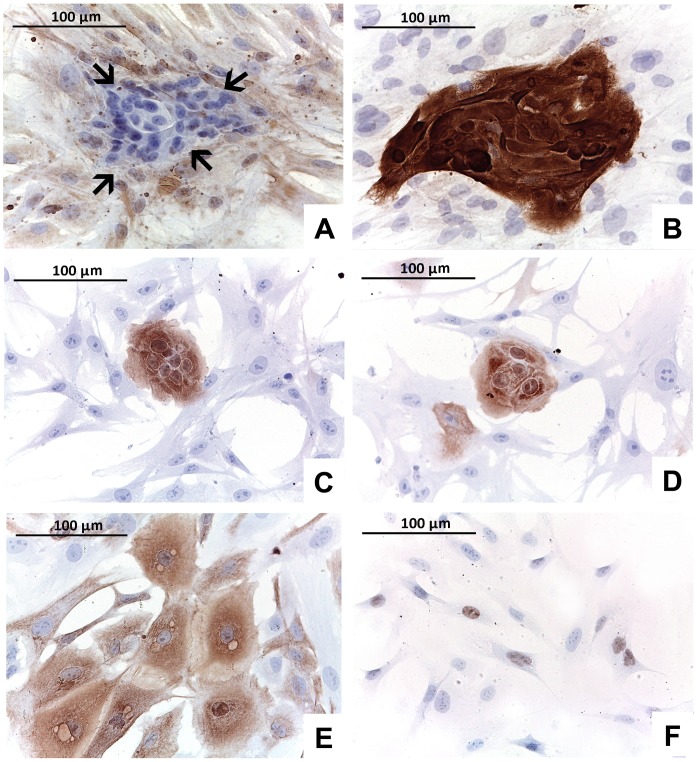
Immunophenotype of cells. A, B: primary culture from an invasive carcinoma of no special type of the breast composed of a mixed population: fibroblasts positive for vimentin (A; arrows indicate epithelial cells) and epithelial cells showing strong cytokeratin-19 positivity (B). C, D: primary culture raised from a lung adenocarcinoma shows epithelial cells positive for cytokeratin-19 (A) and cytokeratin-7 (B). E, F: cells from a primary culture of a Hurtle cell carcinoma of the thyroid show positivity for cytokeratin-19 (E) and TTF1 (F).

The relative percentage of stroma *versus* tumor, as derived from histological slides, seemed not to affect the morphology of cell cultures. In 12 out of 44 viable cultures (27%) the cell population had uniform spindle morphology since the first passage (Table 1). Mixed cell population of spindle and epithelioid cells (Table 1, Figure 3, Figure 4) was observed in the remaining 32 (73%) primary cultures. In cultures derived from adenocarcinomas of breast, lung and colon the epitheliod islets were clearly observed within the spindle components (Figure 4). In 12 out of 32 (37.5%) cases the mixed population could be maintained in culture for three passages. However, in the remaining 20 cases (62.5%) spindle cells that exhibited a great propensity to proliferate *in vitro* overgrew epithelioid cells soon after the first passages (Figure 3). The spindle cell overgrowth was variable across the samples and occurred between passage 2 and passage 5. The results of ICC, performed in primary cultures at first passage, confirmed the phenotype of distinct populations (Figure 4). The mutual exclusive expression of cytokeratins in epithelioid cells and of vimentin in spindle cells suggested the epithelial and stromal nature of cultured cells, respectively (Figure 4). A word of caution for the vimentin positivity in cell cultures should be considered because it is not uncommon to observe vimentin expression in cell cultures of epithelial origin [10], [11], [12]. However in our hand none of the epithelial like cells expressed vimentin, at least at the first passages. Epithelial cells were further characterized by highlighting positivity for tissue-specific cytokeratins, such as cytokeratin-19 for breast and thyroid carcinomas and cytokeratin 7 for lung carcinomas (Figure 4). Cytokeratin-14, studied in breast samples, was expressed neither in the tumor of origin nor in the related cell cultures. TTF1 was instead expressed in thyroid carcinoma and lung adenocarcinomas (Figure 4).

## Discussion

In this feasibility study we report on the setting up of primary cultures from VPAC surgical specimens. By sampling tissue fragments from VPAC preserved specimens we achieved a success rate of approximately 85% leading to a collection of short-term tissue cultures from different neoplastic lesions.

Since the adoption at our institution of an innovative method for tissue collection, transport and storage of surgical specimens based on VPAC technology [5], [7], [8], we have demonstrated in these specimens optimal tissue morphology as well as integrity of antigens for ICC and excellent preservation of nucleic acids to perform molecular analyses [6], [9]. We now show a further implementation of VPAC technology.

The establishment of primary cultures of tumor cells is the goal of many laboratories, however the technique is troublesome, time consuming with a very variable performance rate [1], [13].

When approaching the creation of cell cultures from fresh tumor lesions, collaboration with the pathology laboratory is mandatory. Although the multi-step process involving the creation of a primary culture should stem from a proper tissue handling and sampling, the impact of pre-analytical variables is usually disregarded. In particular, we showed that the surgical time can influence cell viability. In organ transplantation the surgical time may have different definitions and it remains a topic of debate [14], [15], [16]. For cell culture the surgical time starts from the vessel clamping during surgical procedure (corresponding to the loss of perfusion or oxygenation) and ends with the organ excision. It has been shown that significant transcript alterations occur simply as a result of surgical excision [17]. To our knowledge this is the first study that evaluates the effect of the time of surgery on cell viability. We established that the optimal time should be within 1 hour and 30 minutes from the starting of the surgical procedure. During this time the tissue remains alive and is reactive. The temperature of the specimens during surgery decreases very slowly, however disruption of blood flow leads to progressive tissue ischemia and hypoxigenation that cause alterations of cell membrane and receptors, ion regulation, and enzyme systems [18], [19].

The cold ischemia time (which in our study corresponds to the VPAC time) starts when the specimen is excised and ends with incision of tissue and placement in a suitable tissue fixative [18], [19]. Appreciation of the scientific importance of primary tissue handling procedures is growing, particularly for its impact on preservation of nucleic acids and proteins [20], [21], [22], [23], [24]. When dealing with cell cultures and xenograft implantations researchers may ideally wish to collect the sample for experiments directly in the surgery room in order to keep the cold ischemia time as short as possible. However, any such sampling may lead to problems for pathologists in terms of correct gross evaluation of tumor samples (status of surgical margins, staging etc.).

Another issue that should be considered is the optimal temperature to guarantee cell viability. In transplantation pathology it has been shown that a rapid induction of hypothermia at 0–4°C by perfusion of organ with specific solutions better preserves organ viability [25], [26]. We have proposed transfer of surgical specimens vacuum-sealed using a chilled plastic box at 4°C [6], [7]. Our results suggest the avoidance of insulating air around tissues with the VPAC system allows faster cooling at 4°C. From our experience on nucleic acid preservation [7], [9] it seems that it is the prompt cooling that principally influences preservation. This has also been confirmed by independent observations [27] that show that storage at 4°C preserved tissues to a higher degree than storage at room temperature, independently of whether the tissue was subjected to vacuum sealing or not. With this study we prove that VPAC maintains cell viability even after 70 hours, however, as expected, for longer VPAC times, the percentage of cell death increased. Finally, the vacuum sealing in beta-sterilized bags helped prevent bacterial and fungal contamination and consequently, we experienced cell culture infections in only 2% of specimens.

In conclusion, VPAC represented a reliable and reproducible tissue handling protocol for creation of primary cell cultures. Our results also showed how a careful monitoring of surgical and cold ischemia times fostered a good performance of primary tissue cultures. Further studies are needed to more carefully define key parameters governing the reliability of the VPAC method. Nonetheless, the environmentally safe VPAC collection, preservation and storage of surgical specimens already represented a helpful strategy to bridge diagnostic and experimental pathology, offering a new tool for biobanking and improved generation of primary cultures as clinically relevant models of neoplastic lesions.
